# Efficacy of Automated Insulin Delivery Systems in Pediatric Type 1 Diabetes: A Systematic Review of Randomized Controlled Trials

**DOI:** 10.7759/cureus.115014

**Published:** 2026-08-23

**Authors:** Rayan Zakria Mohamed Edris, Thowiba Awad Obaidalla Mohamad, Eman Ali Mohamed Hassan, Ammar Mohammad Suliman Hamza, Maali Shukri AbdElkhier DafaAlla, Samah Dafallah Nimir Ahmed

**Affiliations:** 1 Pediatrics, Leeds Teaching Hospitals NHS Trust, Leeds, GBR; 2 Pediatrics, Alqunfudah General Hospital, Alqunfudah, SAU; 3 Pediatrics, University Hospitals Sussex NHS Foundation Trust, Sussex, GBR; 4 General Pediatric and Child Health, Second Health Cluster in Riyadh, Ministry of Health, Riyadh, SAU; 5 Emergency Medicine, Dr. Suliman Al Habib Medical Group, Riyadh, SAU; 6 Pediatrics, Southend University Hospital NHS Foundation Trust, Essex, GBR

**Keywords:** artificial pancreas, automated insulin delivery, continuous glucose monitoring, hybrid closed-loop, paediatrics, systematic review, time in range, type 1 diabetes

## Abstract

Automated insulin delivery (AID) systems couple continuous glucose monitoring with an algorithm that modulates subcutaneous insulin in real time. The efficacy of these systems in children and adolescents-whose management is complicated by variable insulin requirements, unpredictable food intake, and caregiver dependence-warrants dedicated synthesis.

We systematically searched PubMed/MEDLINE, Embase, Cochrane CENTRAL, Web of Science, and ClinicalTrials.gov to June 30, 2026, for randomized controlled trials comparing AID with non-automated insulin therapy in participants aged 18 years or younger with type 1 diabetes. The protocol specified that, if more than 10 eligible trials were identified, inclusion would be restricted to reports published on or after January 1, 2020; this criterion was subsequently applied. Two reviewers independently screened records, extracted data, and assessed risk of bias using the Cochrane RoB 2 tool. Findings were synthesized narratively without meta-analysis.

Ten trials involving 888 children and adolescents aged 1-18 years met the inclusion criteria. Where time in range was the primary endpoint, AID conferred between-group increases of 8.7 to 12.4 percentage points, equivalent to roughly two to three additional hours in target range daily, with the largest effect observed in children aged 2 to under 6 years. Where HbA1c was the primary endpoint, reductions of 0.32% to 0.5% favored AID, with the greatest absolute benefit among participants furthest from target. Hypoglycemia exposure was not increased. Larger glycemic effects were observed in trials reporting higher device engagement, although this was a between-study observation that was not formally tested. Two trials found no evidence that AID preserved residual beta-cell function, whereas one pilot trial reported more neurotypical brain development with closed-loop therapy. Seven trials were at low risk of bias overall, and three raised some concerns.

AID consistently improves glycemic control without increasing hypoglycemia across the pediatric age spectrum, including preschool children. Sustained engagement, equitable access, and longer-term outcomes remain the principal unresolved issues.

## Introduction and background

Type 1 diabetes is among the most common chronic conditions of childhood, and its global burden is expanding rapidly. Modeling work estimates that 8.4 million people were living with the condition in 2021, with prevalent cases projected to reach between 13.5 and 17.4 million by 2040, and with the steepest relative increases anticipated in resource-limited settings [1]. The therapeutic imperative established by the Diabetes Control and Complications Trial remains unchanged more than three decades later: intensive therapy directed at near-normal glycemia substantially reduces the onset and progression of retinopathy, nephropathy, and neuropathy, at the cost of an increased risk of severe hypoglycemia [2]. In children, this trade-off is particularly consequential, because the developing brain is vulnerable at both extremes of the glycemic range. Longitudinal imaging work has demonstrated that cumulative hyperglycemia in young children with type 1 diabetes is associated with divergent trajectories of gray and white matter development and with lower scores on standardized cognitive testing, with differences widening rather than narrowing over successive years of follow-up [3]. Achieving stable glycemia in pediatric type 1 diabetes is therefore not merely a matter of deferring microvascular complications into adulthood; it is plausibly a determinant of neurodevelopment during the years in which the intervention is delivered.

Against this background, the metrics used to judge success have themselves evolved. The international consensus on time in range established a percentage of time between 70 and 180 mg/dL (3.9-10.0 mmol/L) as a primary continuous glucose monitoring target, recommending that children and adolescents spend more than 70% of the day within this range while limiting time below 70 mg/dL to under 4% [4]. Yet registry data across high-income health systems have consistently shown that a minority of young people meet either these targets or conventional glycated hemoglobin (HbA1c) thresholds using multiple daily injections or non-automated pump therapy.

Automated insulin delivery (AID) systems were developed to close this implementation gap. Conceptually, an AID system has three components that operate as a single loop: a continuous glucose monitor that reports interstitial glucose every one to five minutes; an insulin pump that delivers rapid-acting insulin subcutaneously; and a control algorithm, resident on the pump or on a paired smartphone, that reads each new glucose value, projects glucose forward over the coming hours, and adjusts insulin delivery accordingly. This adjustment is what distinguishes AID from the therapies that preceded it. Sensor-augmented pump therapy displays glucose data but leaves every dosing decision to the user, and predictive low-glucose suspend systems act only in one direction, pausing insulin when hypoglycemia is anticipated. An AID algorithm instead modulates delivery continuously in both directions, increasing basal rates or giving small automatic correction doses when glucose is rising and reducing or suspending delivery when it is falling. Almost all systems in current clinical use are described as hybrid rather than fully closed-loop, because the user must still announce meals and count carbohydrates for mealtime doses; the algorithm governs background insulin and corrections between meals and overnight. Successive international guidelines now position these systems as the preferred insulin delivery modality for children and adolescents wherever they are available [5]. The pediatric context imposes distinctive demands on them: total daily insulin doses may be very small, carbohydrate intake and physical activity are erratic, day-to-day insulin requirements vary more widely than in adults, and the burden of operating the technology falls substantially on parents, school staff, and other caregivers rather than on the person with diabetes [6].

Consensus statements have synthesized the AID evidence base across the lifespan and have concluded that these systems are safe and effective, but such statements necessarily aggregate populations that differ substantially in physiology and in the practical determinants of device use [7]. Much of the pooled evidence derives from trials in adults or in mixed adult-pediatric cohorts, and the quantitative syntheses that have addressed children have generally prioritized pooled effect estimates for time in range over a careful appraisal of how efficacy varies with age, baseline glycemia, device engagement, and trial design, and over the non-glycemic outcomes that arguably matter most to families. A synthesis restricted to randomized evidence generated exclusively in participants aged 18 years or younger, and one that examines beta-cell, neurocognitive, and psychosocial endpoints alongside conventional glycemic measures, is therefore warranted. The present systematic review was undertaken to determine the efficacy and safety of AID systems compared with non-automated insulin therapy in children and adolescents with type 1 diabetes, and to characterize the factors that may modify the magnitude of benefit.

## Review

Materials and methods

Study Design and Protocol

This systematic review was conducted and reported in accordance with the Preferred Reporting Items for Systematic Reviews and Meta-Analyses (PRISMA) 2020 statement [8]. The review question, eligibility criteria, search strategy, data items, and synthesis plan were specified in a written protocol before the formal searches were conducted, including the conditional recency criterion described below. The protocol was not registered in PROSPERO or another public registry, and we acknowledge this as a limitation of the review; the protocol document and all data extraction forms are available from the corresponding author on request. As the review analyzed only previously published, de-identified aggregate data, ethical approval and informed consent were not required.

Eligibility Criteria

Eligibility was framed using the population, intervention, comparator, outcomes, and study design (PICOS) structure and is presented in full in Table 1. In brief, we included randomized controlled trials with a parallel-group or crossover design that compared any AID system with non-automated insulin therapy in participants aged 18 years or younger with type 1 diabetes, and that reported at least one predefined glycemic or safety outcome over an intervention period of at least two weeks. Trials enrolling mixed adult-pediatric cohorts were excluded unless outcomes for participants aged 18 years or younger were reported separately, and comparisons of one AID system against another were excluded because such trials do not address the review question. Reviews, meta-analyses, conference abstracts, editorials, letters, protocols, preprints, non-randomized and single-arm studies, and secondary or duplicate reports of already-included trials were excluded.

**Table 1:** Eligibility criteria according to the PICOS framework. PICOS: population, intervention, comparator, outcomes, study design; AID: automated insulin delivery; CGM: continuous glucose monitoring; HbA1c: glycated hemoglobin; mg/dL: milligrams per deciliter; mmol/L: millimoles per liter. ≤: less than or equal to; –: to (denoting a range).

PICOS domain	Inclusion criteria	Exclusion criteria
Population (P)	Children and adolescents aged ≤ 18 years at enrollment with a clinical diagnosis of type 1 diabetes, irrespective of diabetes duration, baseline glycated hemoglobin (HbA1c), or prior insulin delivery modality; trials of newly diagnosed participants were eligible.	Trials enrolling adults, or mixed adult–pediatric cohorts in which outcomes for participants aged ≤ 18 years were not reported separately; type 2, monogenic, cystic fibrosis-related, or steroid-induced diabetes.
Intervention (I)	Any automated insulin delivery (AID) system, defined as an insulin pump coupled to a continuous glucose monitor (CGM) and a control algorithm that automatically modulates subcutaneous insulin delivery in real time (hybrid, advanced hybrid, or fully closed-loop configurations), used in an outpatient or free-living setting.	Sensor-augmented pump therapy without algorithmic dose modulation; threshold-suspend or predictive low-glucose suspend used as the intervention arm; overnight-only or single-session inpatient closed-loop; do-it-yourself systems without regulatory-grade evaluation.
Comparator (C)	Non-automated insulin therapy, comprising multiple daily injections with or without CGM, continuous subcutaneous insulin infusion, sensor-augmented pump therapy, predictive low-glucose suspend, or usual/standard diabetes care.	Comparison of one AID system against another AID system, or of algorithm, insulin, or meal-announcement variants within the same AID platform.
Outcomes (O)	Primary: percentage of time in range 70–180 mg/dL (3.9–10.0 mmol/L) and HbA1c. Secondary: time above and below range, mean sensor glucose, glycemic variability, severe hypoglycemia, diabetic ketoacidosis, system engagement, and non-glycemic outcomes including residual beta-cell function, neurocognitive and psychosocial measures.	Studies reporting none of the specified glycemic or safety outcomes; modeling, cost-effectiveness, or in-silico analyses reporting no primary participant-level data.
Study design (S)	Randomized controlled trials with a parallel-group or crossover design, of at least two weeks' intervention duration, published as full-text, peer-reviewed original articles in English between January 1, 2020 and June 30, 2026.	Narrative and systematic reviews, meta-analyses, conference abstracts, editorials, commentaries, letters, case reports, study protocols, preprints, non-randomized and single-arm studies, and secondary or duplicate reports of trials already included.

A preliminary search without date restriction identified more than 10 trials meeting the clinical eligibility criteria, including several published between 2016 and 2019. In accordance with the prespecified protocol, a recency restriction was therefore applied and only reports published on or after January 1, 2020 were retained, on the grounds that trials predating this threshold overwhelmingly evaluated first-generation hybrid closed-loop platforms and adjunctive, calibration-dependent sensors that no longer reflect contemporary practice.

Information Sources and Search Strategy

We searched PubMed/MEDLINE, Embase, the Cochrane Central Register of Controlled Trials, and the Web of Science Core Collection from inception to June 30, 2026, supplemented by a search of ClinicalTrials.gov to identify completed trials with linked publications. The strategy combined controlled vocabulary and free-text terms for the intervention (closed-loop, artificial pancreas, automated insulin delivery, artificial beta cell), the condition (type 1 diabetes), and the population (child, adolescent, pediatric, youth, infant), restricted by a randomized trial filter. The complete executable search string for each source, together with the field codes, controlled vocabulary, limits applied, and the date on which each search was run, is reproduced in Table 2 so that the search can be reproduced exactly. Reference lists of all included trials and of recent systematic reviews were hand-searched, and forward citation tracking was performed for each included report.

**Table 2:** Executed search strategy. Searches were limited to English-language records. No publication-date filter was applied at the search stage; the recency restriction was applied during full-text screening. NLM: National Library of Medicine; MEDLINE: Medical Literature Analysis and Retrieval System Online; CENTRAL: Cochrane Central Register of Controlled Trials; MeSH: Medical Subject Headings; Emtree: Embase subject headings; exp: exploded (term searched together with its narrower terms); ti,ab: title or abstract field; ti,ab,kw: title, abstract, or keyword field; TS: topic search. *: truncation operator retrieving all word endings; #: search set number; —: not applicable.

Database or register (platform)	Date searched	Records retrieved	Complete search string, field codes, and limits applied
PubMed/MEDLINE (NLM)	June 30, 2026	328	("closed-loop"[tiab] OR "closed loop"[tiab] OR "artificial pancreas"[tiab] OR "automated insulin delivery"[tiab] OR "artificial beta cell"[tiab] OR "Pancreas, Artificial"[MeSH Terms]) AND ("Diabetes Mellitus, Type 1"[MeSH Terms] OR "type 1 diabetes"[tiab]) AND (child*[tiab] OR paediatric[tiab] OR pediatric[tiab] OR adolescen*[tiab] OR youth[tiab] OR infant*[tiab] OR "Child"[MeSH Terms] OR "Adolescent"[MeSH Terms]) AND (random*[tiab] OR trial[tiab]). Limits: English language. No publication-date limit applied at the search stage.
Embase (Elsevier, Embase.com)	June 30, 2026	169	('closed loop insulin delivery'/exp OR 'artificial pancreas'/exp OR 'automated insulin delivery':ti,ab OR 'artificial beta cell':ti,ab) AND 'insulin dependent diabetes mellitus'/exp AND (child:ti,ab OR adolescent:ti,ab OR paediatric:ti,ab OR pediatric:ti,ab OR youth:ti,ab) AND 'randomized controlled trial'/exp AND [english]/lim. Field codes: /exp = exploded Emtree term; :ti,ab = title or abstract.
Cochrane CENTRAL (Wiley)	June 30, 2026	138	#1 MeSH descriptor: [Pancreas, Artificial] explode all trees; #2 ("closed loop" OR "artificial pancreas" OR "automated insulin delivery"):ti,ab,kw; #3 MeSH descriptor: [Diabetes Mellitus, Type 1] explode all trees; #4 ("type 1 diabetes"):ti,ab,kw; #5 (child* OR adolescen* OR paediatric OR pediatric OR youth):ti,ab,kw; #6 (#1 OR #2) AND (#3 OR #4) AND #5. Limits: Trials records only.
Web of Science Core Collection (Clarivate)	June 30, 2026	213	TS=(("closed-loop" OR "artificial pancreas" OR "automated insulin delivery") AND "type 1 diabetes" AND (child* OR adolescen* OR pediatric OR paediatric OR youth) AND random*). Limits: document type = Article; language = English. Field code: TS = topic (title, abstract, author keywords, Keywords Plus).
ClinicalTrials.gov	June 30, 2026	93	Condition or disease: type 1 diabetes. Intervention: closed-loop OR automated insulin delivery. Age: Child (birth to 17 years). Study type: Interventional, with randomized allocation. Status: completed studies with posted results or an identifiable linked publication.
Total	—	941	All sources searched from inception to June 30, 2026. Reference lists of included trials and of recent systematic reviews were hand-searched, and forward citation tracking was performed for each included report.

Study Selection

All records were imported into reference management software and deduplicated. Two reviewers independently screened titles and abstracts against the eligibility criteria, and then independently assessed the full texts of all reports retained after title and abstract screening. Disagreements at either stage were resolved by discussion, with recourse to a third reviewer where consensus could not be reached. Reasons for exclusion were recorded for every full-text report and are summarized in the PRISMA flow diagram; the complete study-level list of excluded full-text reports with reasons is available from the corresponding author on request, and the reports whose exclusion is most likely to affect interpretation are tabulated in the Results section.

Data Extraction

Data were extracted independently and in duplicate using a piloted, standardized form. Extracted items comprised first author, year of publication, country and number of centers, trial design and allocation ratio, intervention duration, eligibility criteria, numbers randomized and analyzed, participant age and baseline glycemic characteristics, the AID system and its algorithm, the comparator regimen, primary and secondary outcomes with corresponding effect estimates and measures of precision, device engagement, adverse events including severe hypoglycemia and diabetic ketoacidosis, and funding sources. Where a trial reported both intention-to-treat and per-protocol analyses, the intention-to-treat estimate was extracted. Discrepancies were resolved by consensus against the source publication.

Risk-of-Bias Assessment

Risk of bias was appraised independently by two reviewers using the revised Cochrane risk-of-bias tool for randomized trials (RoB 2), across the domains of randomization process, deviations from intended interventions, missing outcome data, measurement of the outcome, and selection of the reported result [9]. Each domain was rated as low risk, some concerns, or high risk, and an overall judgment was derived using the algorithm specified by the tool. In accordance with RoB 2 guidance, assessment was anchored to a single prespecified result in each trial, namely the trial’s own primary endpoint: time in range for four trials, HbA1c for two trials, time below range for one trial, stimulated C-peptide area under the curve for two trials, and brain magnetic resonance imaging metrics for one trial. In every case, the effect of assignment to the intervention was the effect of interest, and the signaling questions were answered against the intention-to-treat analysis. Because open-label allocation is unavoidable when a visible medical device is compared with conventional therapy, the domain concerning deviations from intended interventions was assessed with explicit attention to whether the effect of assignment to the intervention was estimated using an appropriate intention-to-treat analysis, rather than penalizing the absence of blinding per se. Domain-level and overall judgments were visualized as a traffic-light plot using the robvis tool [10]. Completed signaling-question worksheets are available from the corresponding author on request.

Data Synthesis

We judged statistical pooling inappropriate and therefore performed no meta-analysis. This decision did not rest on statistical heterogeneity alone, which would not by itself preclude a random-effects meta-analysis, but on the absence of a common estimand across the included trials. The trials did not measure the same quantity: only four reported time in range as a primary endpoint with a confidence interval, two reported HbA1c, two reported stimulated C-peptide, one reported time below range under a non-inferiority framework, and one reported brain imaging metrics. They also spanned age strata from infancy to late adolescence-groups in which the comparator therapy, the caregiver role, and the achievable target differ materially-five distinct control algorithms, four different comparator regimens, both parallel-group and crossover designs whose effect estimates are not directly interchangeable, and intervention durations from six weeks to 24 months. Pooling across these dimensions would have produced a summary estimate that corresponded to no answerable clinical question.

We therefore synthesized the results narratively and tabulated them by outcome domain, following the principles of Synthesis Without Meta-analysis (SWiM) reporting [11]. Trials were first grouped by primary outcome domain (glycemic efficacy, safety and engagement, and non-glycemic outcomes); within the glycemic domain they were ordered by the metric serving as the primary endpoint, and effect estimates were extracted with their reported measures of precision and direction relative to the comparator. Vote counting on the basis of statistical significance was not used. Between-group effect estimates for time in range are displayed graphically for the subset of trials in which this metric was the primary endpoint and a confidence interval was reported, with no pooled estimate calculated and no summary diamond plotted. Percentage-point differences are reported throughout as pp; HbA1c is reported in the units used by the source publication, with conversion where the source reported both, and all effect estimates are accompanied by 95% confidence intervals where these were available. Observed differences in effect size across age strata or across levels of device engagement are reported as between-study observations; no formal subgroup interaction test or meta-regression was undertaken, and these observations are therefore treated as hypothesis-generating rather than as established effect modification.

Results

Study Selection

**Figure 1:**
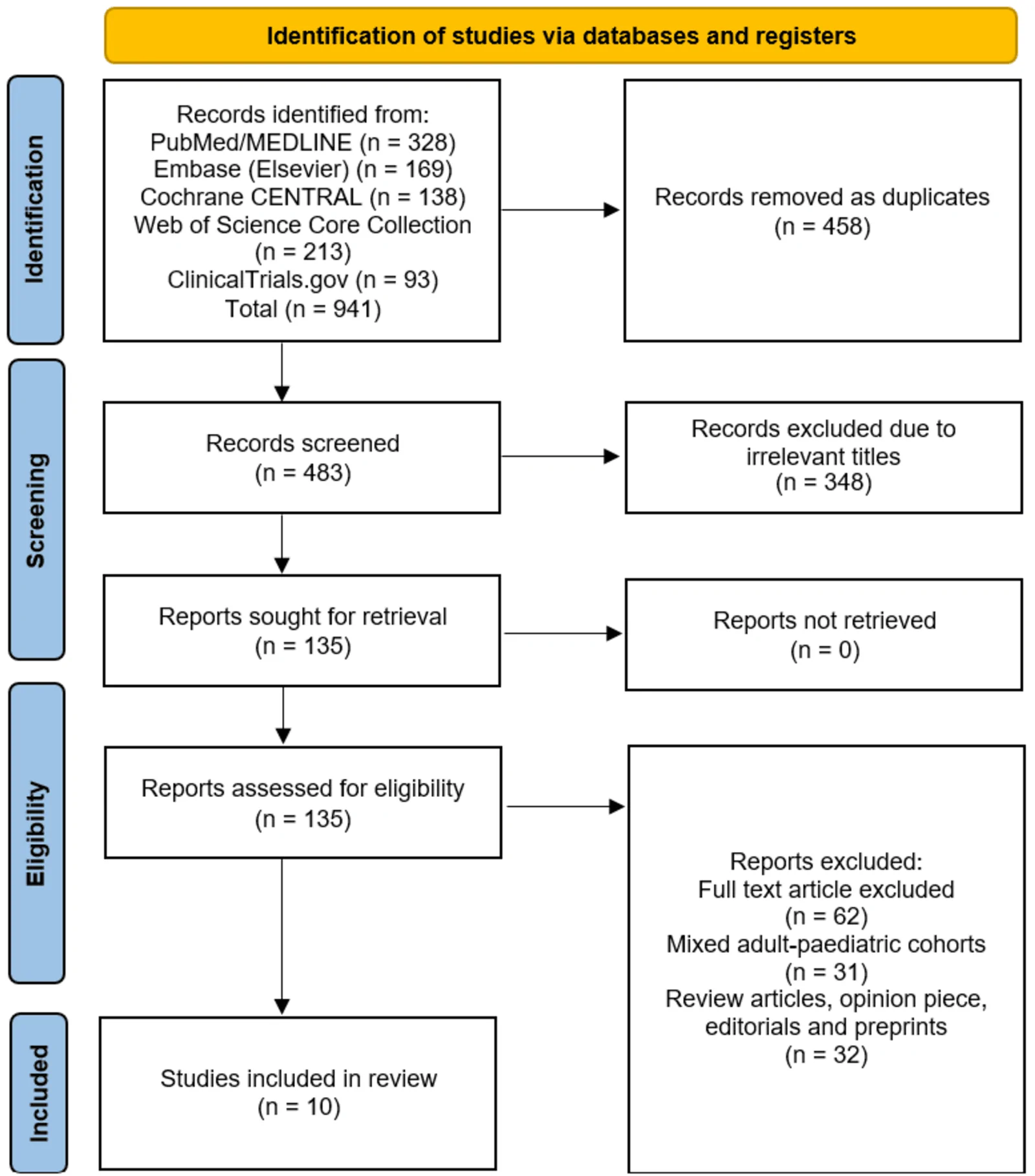
PRISMA 2020 flow diagram of study identification, screening and inclusion. All reports sought for retrieval were successfully obtained. PRISMA: Preferred Reporting Items for Systematic Reviews and Meta-Analyses; MEDLINE: Medical Literature Analysis and Retrieval System Online; CENTRAL: Cochrane Central Register of Controlled Trials; n: number of records or reports. =: equals.

The searches retrieved 941 records: 328 from PubMed/MEDLINE, 169 from Embase, 138 from the Cochrane Central Register of Controlled Trials, 213 from the Web of Science Core Collection, and 93 from ClinicalTrials.gov. After 458 duplicates were removed, 483 records were screened by title and abstract, and 348 were excluded as irrelevant. The remaining 135 reports were sought for retrieval, and all were obtained. Of the 135 reports assessed for eligibility, 125 were excluded: 62 did not satisfy the full eligibility criteria on full-text review, 31 reported mixed adult-pediatric cohorts without separately reported pediatric data, and 32 were reviews, opinion pieces, editorials, or preprints. Ten randomized controlled trials involving 888 children and adolescents were included in the qualitative synthesis [12-21]. The selection process is summarized in Figure 1.

Four excluded reports warrant explicit mention because they are randomized trials of AID in young people that readers may expect to see included, and because the reason for their exclusion bears directly on the interpretation of this review. These are summarized in Table 3. Three were excluded because they enrolled mixed adolescent-young adult cohorts extending to 25 or 29 years of age without reporting outcomes separately for participants aged 18 years or younger [22-24], and one was excluded because its comparator was a different activation schedule of the same AID system rather than non-automated insulin therapy [25]. All four are considered in the Discussion as adjacent evidence.

**Table 3:** Randomized trials of automated insulin delivery in young people excluded at full-text review, with reasons. AID: automated insulin delivery; HbA1c: glycated hemoglobin; FLAIR: Fuzzy Logic Automated Insulin Regulation. ≤: less than or equal to; >: greater than; –: to (denoting a range).

Report	Design and population	Primary reason for exclusion	Comment
Bergenstal et al., 2021 (FLAIR) [22]	Multicenter randomized crossover trial; 113 participants aged 14–29 years	Mixed adolescent–adult cohort; outcomes for participants aged ≤ 18 years not reported separately	Also compared one AID system with another AID system rather than with non-automated therapy; discussed as adjacent evidence
Abraham et al., 2021 [23]	Multicenter randomized controlled trial; 135 participants aged 12–25 years	Mixed adolescent–young adult cohort without separately reported data for those aged ≤ 18 years	Reported glycemic and psychosocial outcomes concordant with the present synthesis; discussed as adjacent evidence
Abraham et al., 2025 [24]	Multicenter randomized clinical trial; 6 months; youth aged 12–25 years with HbA1c > 8.5%	Mixed adolescent–young adult cohort without separately reported data for those aged ≤ 18 years	Identified in the updated search to June 30, 2026; the largest recent trial in youth with elevated HbA1c and discussed in detail
Renard et al., 2022 [25]	Multicenter randomized trial; 122 prepubertal children	Comparator was another activation schedule of the same AID system rather than non-automated insulin therapy	Population would otherwise have been eligible; excluded solely on the comparator criterion

Characteristics of the Included Trials

**Figure 2:**
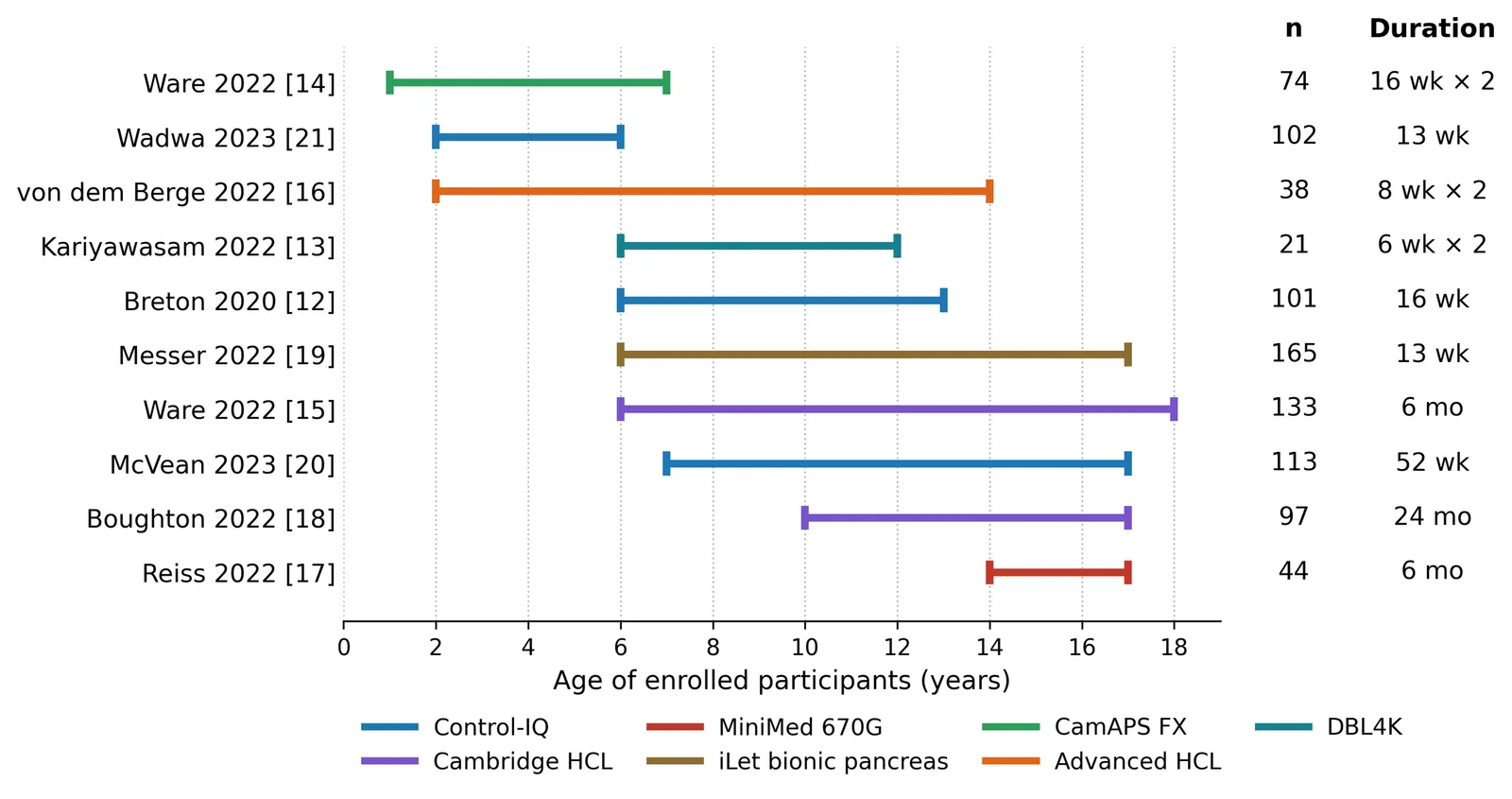
Evidence map of the included trials by enrolled age range, intervention duration and sample size. Horizontal bars span the age range of participants enrolled in each trial and are colored by the automated insulin delivery system evaluated; the adjacent columns give the number randomized and the intervention duration. For crossover trials, the duration shown is per treatment period. Control-IQ (Tandem Diabetes Care, Inc., San Diego, CA, USA), CamAPS FX (CamDiab Ltd, London, UK), DBL4K (Diabeloop SA, Grenoble, France), MiniMed 670G (Medtronic, Inc., Minneapolis, MN, USA), and iLet (Beta Bionics, Inc., Irvine, CA, USA) are proprietary names of the automated insulin delivery systems evaluated. HCL: hybrid closed loop; n: number of participants randomized; wk: weeks; mo: months. ×: multiplied by (indicating the number of treatment periods in a crossover trial).

The included trials were published between 2020 and 2023 and were conducted in the United States, the United Kingdom, France, Belgium, Germany, Austria, and Luxembourg [12-21]. Six used a parallel-group design and four a crossover design. Sample sizes ranged from 21 to 165 participants, and intervention durations ranged from a six-week home phase per period to 24 months. The evaluated systems comprised the t:slim X2 with Control-IQ (Tandem Diabetes Care, Inc., San Diego, CA, USA) [12,20,21], the Diabeloop DBL4K platform (Diabeloop SA, Grenoble, France) [13], the Cambridge algorithm in its CamAPS FX configuration (CamDiab Ltd, London, UK) and FlorenceM configuration (University of Cambridge, Cambridge, UK) [14,15,18], a Medtronic advanced hybrid closed-loop system (Medtronic, Inc., Minneapolis, MN, USA) [16], the MiniMed 670G (Medtronic, Inc., Minneapolis, MN, USA) [17], and the insulin-only iLet bionic pancreas (Beta Bionics, Inc., Irvine, CA, USA) [19]. Comparators were sensor-augmented pump therapy [12-14,16], usual care with pump therapy [15], standard care comprising multiple daily injections or a non-automated pump with or without continuous glucose monitoring [17,19-21], and multiple daily injections in newly diagnosed participants [18]. Full characteristics are presented in Table 4, and the distribution of trials across the pediatric age spectrum is displayed in Figure 2.

**Table 4:** Characteristics of the included randomized controlled trials. RCT: randomized controlled trial; T1D: type 1 diabetes; AID: automated insulin delivery; CGM: continuous glucose monitoring; HbA1c: glycated hemoglobin; TIR: time in range; TBR: time below range; TAR: time above range; DKA: diabetic ketoacidosis; AUC: area under the curve; CI: confidence interval; IQ: intelligence quotient; MRI: magnetic resonance imaging; SAP: sensor-augmented pump; pp: percentage points; n: number of participants; y: years; h/day: hours per day; mg/dL: milligrams per deciliter; mmol/mol: millimoles per mole; d: Cohen’s d (standardized effect size); P: probability value. ±: plus or minus; −: minus (denoting a reduction); –: to (denoting a range); ≤: less than or equal to; ≥: greater than or equal to; <: less than; >: greater than; %: percent.

Study	Country	Design	Population	Age range (mean)	n randomized	Intervention	Comparator	Outcomes assessed	Key findings
Breton et al., 2020 [12]	USA	Multicenter, open-label, parallel-group RCT (3:1); 16 weeks	Children with established T1D; baseline HbA1c 5.7–10.1%	6–13 y (11.3 y)	101 (78/23)	t:slim X2 with Control-IQ hybrid closed-loop	Sensor-augmented pump ± predictive low-glucose suspend	TIR (primary), TBR, TAR, mean glucose, HbA1c, safety	TIR rose from 53±17% to 67±10% with AID vs 51±16% to 55±13% with control; mean adjusted difference 11 pp (95% CI 7–14; P<0.001). No DKA or severe hypoglycemia.
Kariyawasam et al., 2022 [13]	France, Belgium	Multicenter, open-label, randomized, two-period crossover non-inferiority trial; 72-h inpatient plus 6-week home phase per period	Prepubertal (Tanner stage I) children with T1D ≥1 y and HbA1c ≤9%	6–12 y	21	Diabeloop DBL4K hybrid closed-loop (Kaleido pump, Dexcom G6)	Sensor-augmented pump (open loop)	Time in hypoglycemia <70 mg/dL during the inpatient phase (primary, non-inferiority margin −2.5%), TIR, technical performance, safety	Time in hypoglycemia was lower with AID (2.04%, 95% CI 0.44–3.64) than open loop (7.06%, 95% CI 5.46–8.66; one-sided P<0.0001). No severe hypoglycemia or ketoacidosis.
Ware et al., 2022 [14]	UK, Austria, Germany, Luxembourg	Multicenter, open-label, randomized crossover trial; two 16-week periods	Very young children with T1D; baseline HbA1c 7.3±0.7%	1–7 y (5.6 y)	74	CamAPS FX hybrid closed-loop (Cambridge algorithm)	Sensor-augmented pump therapy	TIR (primary), TAR, HbA1c, mean sensor glucose, TBR, safety	TIR was 8.7 pp higher with AID (95% CI 7.4–9.9; P<0.001); TAR −8.5 pp, HbA1c −0.4 pp, and mean sensor glucose −12.3 mg/dL, all favoring AID. Hypoglycemia exposure was unchanged (P=0.74).
Ware et al., 2022 [15]	UK, USA	Multicenter, multinational, parallel-group RCT; 6 months	Pump-treated children and adolescents with screening HbA1c 7.0–10.0%	6–18 y (13.0 y)	133 (65/68)	Cambridge closed-loop algorithm (FlorenceM or CamAPS FX configuration)	Usual care with insulin pump therapy ± CGM	HbA1c change at 6 months (primary), CGM metrics, closed-loop utilization, safety	HbA1c was lower with AID (between-group difference −3.5 mmol/mol, 95% CI −6.5 to −0.5; −0.32 pp, −0.59 to −0.04; P=0.023). Utilization was 40% with FlorenceM but 93% with CamAPS FX, attenuating overall efficacy.
von dem Berge et al., 2022 [16]	Germany	Single-center, randomized, controlled, two-phase crossover trial; 8 weeks per phase	Preschool and school-aged children with T1D on pump therapy	2–14 y (two strata: 2–6 y and 7–14 y)	38	Advanced hybrid closed-loop (Auto Mode)	Sensor-augmented pump and predictive low-glucose management	TIR (primary), CGM metrics, HbA1c, DISABKIDS quality of life, hypoglycemia fear, safety	Mean TIR reached 73±6% in preschoolers (+8 pp vs SAP) and 69±8% in school children (+15 pp vs SAP); the >70% TIR target was met by 88% and 50% respectively. HbA1c fell from 7.4±0.9% to 6.9±0.5% (P=0.0002).
Reiss et al., 2022 [17]	USA	Multicenter, parallel-group, assessor-blinded pilot RCT; 6 months	Adolescents with T1D diagnosed before 8 years of age	14–17 y	44 (42 analyzed)	MiniMed 670G hybrid closed-loop with 24-h sensor-augmented therapy	Standard care (multiple daily injections or open-loop pump)	Structural and functional brain MRI metrics (primary), standardized IQ, CGM metrics, safety	AID produced greater normalization of cortical surface area (d=0.74; P=0.018), caudate volume (d=0.75; P=0.018), and white-matter fractional anisotropy (d=0.68; P=0.030), and higher Perceptual Reasoning Index scores (d=0.82; P=0.009), alongside reduced hyperglycemia and glycemic variability.
Boughton et al., 2022 [18]	UK	Multicenter, open-label, parallel-group RCT; 24 months	Youth randomized within 21 days of T1D diagnosis	10.0–16.9 y (12 y)	97 (51/46)	Cambridge hybrid closed-loop therapy	Standard insulin therapy (predominantly multiple daily injections)	Stimulated C-peptide AUC at 12 months (primary), TIR, HbA1c, TBR, safety, quality of life	C-peptide AUC did not differ (0.35 vs 0.46 pmol/mL; adjusted difference −0.06, 95% CI −0.14 to 0.03; P=0.19), but TIR was 10 pp higher (64±14% vs 54±23%; 95% CI 2–17) and HbA1c 0.4% lower with AID. Median closed-loop use was 66%.
Messer et al., 2022 [19]	USA	Multicenter, parallel-group RCT (2:1); 13 weeks	Youth with T1D across a wide baseline HbA1c range (5.8–12.2%); 35% previously on injections	6–17 y	165 (112/53)	iLet insulin-only bionic pancreas	Personal standard-care insulin delivery plus real-time CGM	HbA1c at 13 weeks (primary), TIR, TBR, mean CGM glucose, safety	HbA1c fell from 8.1±1.2% to 7.5±0.7% with AID versus 7.8±1.1% unchanged with standard care (adjusted difference −0.5%, 95% CI −0.7 to −0.2; P<0.001); TIR increased by 10 pp (2.4 h/day). Participants with baseline HbA1c ≥9.0% improved from 9.7±0.8% to 7.9±0.6%.
McVean et al., 2023 [20]	USA	Multicenter, randomized clinical trial (factorial design); 52 weeks	Youth with newly diagnosed stage 3 T1D randomized within 31 days of diagnosis	7–17 y	113	Intensive diabetes management incorporating Control-IQ automated insulin delivery	Standard diabetes care with continuous glucose monitoring	Stimulated C-peptide AUC at 52 weeks (primary), TIR, HbA1c, safety	Near-normalization of glucose did not preserve beta-cell function: C-peptide did not differ significantly between groups at 52 weeks. Mean TIR at 52 weeks was 78% with intensive AID-based management versus 64% with standard care.
Wadwa et al., 2023 [21]	USA	Multicenter, open-label, parallel-group RCT (2:1); 13 weeks	Very young children with T1D; baseline HbA1c 5.2–11.5%; 35% previously on injections	2 to <6 y	102 (68/34)	t:slim X2 with Control-IQ hybrid closed-loop, initiated virtually in 81%	Standard care (insulin pump or multiple daily injections) plus CGM	TIR (primary), TAR >250 mg/dL, TBR, mean glucose, HbA1c, safety	TIR increased from 56.7±18.0% to 69.3±11.1% with AID versus 54.9±14.7% to 55.9±12.6% with standard care (mean adjusted difference 12.4 pp, 95% CI 9.5–15.3; P<0.001), equivalent to approximately 3 additional hours in range per day.

Glycemic Efficacy

**Figure 3:**
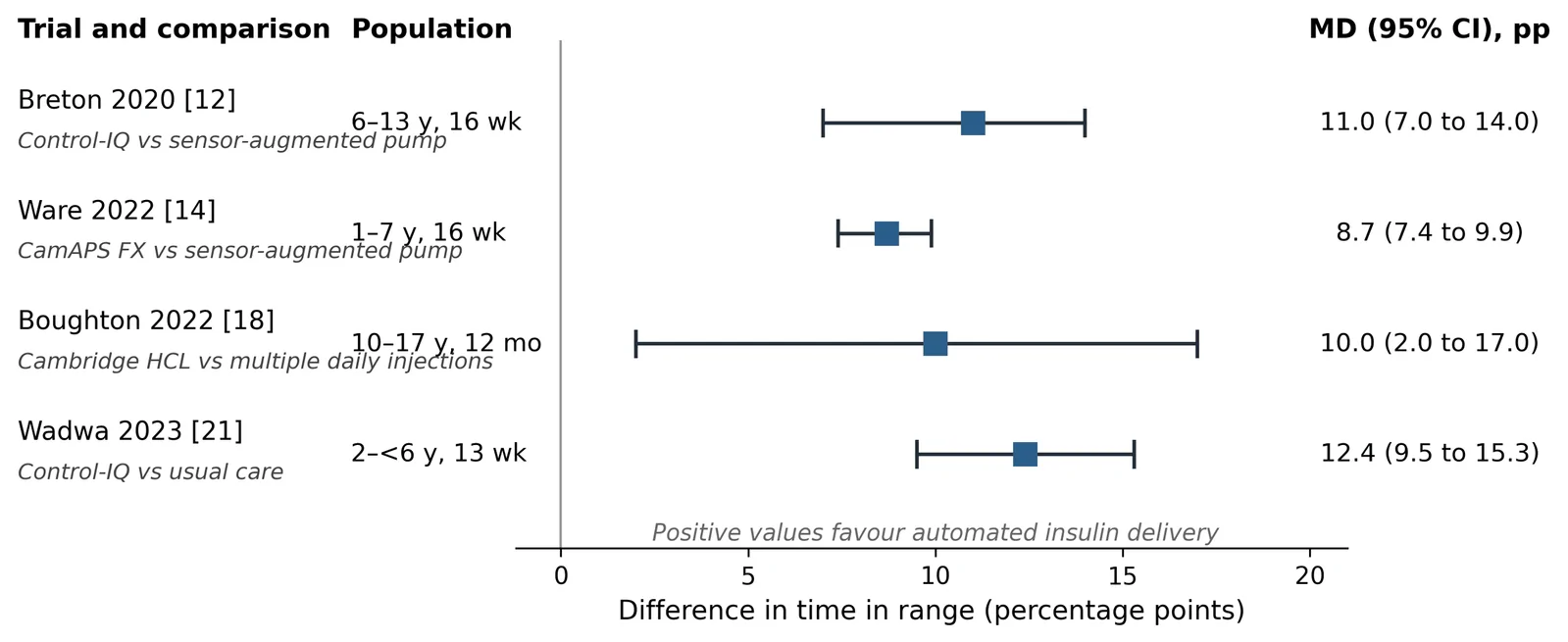
Between-group differences in time in range 70–180 mg/dL reported by the included trials. Squares denote point estimates and horizontal lines the 95% confidence intervals. Estimates are displayed individually; no statistical pooling was performed because the included trials did not share a common estimand. Control-IQ (Tandem Diabetes Care, Inc., San Diego, CA, USA) and CamAPS FX (CamDiab Ltd, London, UK) are proprietary names of the automated insulin delivery systems evaluated; the Cambridge hybrid closed-loop algorithm was developed at the University of Cambridge, Cambridge, UK. MD: mean difference; CI: confidence interval; pp: percentage points; HCL: hybrid closed loop; wk: weeks; mo: months; y: years; mg/dL: milligrams per deciliter. –: to (denoting a range); <: less than; %: percent.

Time in range was the designated primary endpoint in three trials, all of which demonstrated a statistically significant and clinically substantial advantage for AID. In children aged 6 to 13 years, 16 weeks of Control-IQ therapy raised mean time in range from 53±17% to 67±10%, compared with a rise from 51±16% to 55±13% under sensor-augmented pump therapy, a mean adjusted difference of 11 pp (95% CI 7-14; P<0.001) corresponding to 2.6 additional hours in range each day [12]. In very young children aged 1 to 7 years, a crossover comparison of the CamAPS FX system with sensor-augmented pump therapy over two 16-week periods yielded a between-treatment difference of 8.7 pp (95% CI 7.4-9.9; P<0.001), accompanied by an 8.5 pp reduction in time above 180 mg/dL, a 0.4 pp reduction in HbA1c, and a 12.3 mg/dL reduction in mean sensor glucose [14]. The largest effect was observed in the youngest cohort studied: among children aged 2 to under 6 years, 13 weeks of Control-IQ therapy increased time in range from 56.7±18.0% to 69.3±11.1%, against essentially no change under standard care (54.9±14.7% to 55.9±12.6%), a mean adjusted difference of 12.4 pp (95% CI 9.5-15.3; P<0.001), equivalent to approximately three additional hours in range daily [21]. These estimates, together with the time-in-range difference reported as a key secondary endpoint in newly diagnosed adolescents [18], are displayed in Figure 3.

Where HbA1c served as the primary endpoint, the direction of effect was concordant, but the magnitude was more modest and, across trials, appeared more closely related to device engagement. In a six-month parallel-group trial in 133 pump-treated children and adolescents aged 6 to 18 years, the Cambridge algorithm reduced HbA1c by 3.5 mmol/mol relative to usual care (95% CI −6.5 to −0.5; equivalent to −0.32 pp, 95% CI −0.59 to −0.04; P=0.023) [15]. The authors attributed the attenuated effect to markedly unequal utilization between the two hardware configurations tested, with median closed-loop use of only 40% (IQR 26-53) using the FlorenceM enclosure compared with 93% (IQR 88-96) using CamAPS FX. A larger HbA1c effect was recorded in a 13-week trial of the insulin-only bionic pancreas in 165 youth aged 6 to 17 years, in which HbA1c fell from 8.1±1.2% to 7.5±0.7% with the device while remaining unchanged at 7.8±1.1% under standard care, an adjusted difference of −0.5% (95% CI −0.7 to −0.2; P<0.001), alongside a 10 pp increase in time in range [19]. The gradient of benefit by baseline control was pronounced: among the 34 participants with a baseline HbA1c of 9.0% or above, HbA1c fell to 7.9±0.6% with the bionic pancreas while the corresponding standard-care subgroup remained at 9.8±0.8%.

Two crossover trials extended these findings across a broader pediatric age range using more demanding comparators. A single-center trial in 38 children aged 2 to 14 years compared eight weeks of hybrid closed-loop therapy with both sensor-augmented pump therapy and predictive low-glucose management, and found mean time in range of 73±6% in preschoolers and 69±8% in school-aged children during closed-loop use, representing gains of 8 pp and 15 pp respectively over sensor-augmented pump therapy and of 6 pp and 14 pp over predictive low-glucose management [16]. The consensus target of spending more than 70% of time in range was achieved by 88% of preschoolers and 50% of school-aged children, and overall HbA1c fell from 7.4±0.9% to 6.9±0.5% (P=0.0002). Notably, this trial demonstrated incremental benefit even relative to an already automated suspend-based comparator, indicating that the advantage of AID is not attributable simply to concurrent sensor use. In the smallest included trial, 21 prepubertal children aged 6 to 12 years participated in a crossover trial comparing the DBL4K system with sensor-augmented pump therapy in which protection from hypoglycemia was the prespecified primary endpoint; time below 70 mg/dL during the inpatient phase was 2.04% (95% CI 0.44-3.64) with closed-loop compared with 7.06% (95% CI 5.46-8.66) with open loop, satisfying the non-inferiority margin of −2.5% and demonstrating superiority (one-sided P<0.0001) [13]. Detailed glycemic outcomes for all ten trials are set out in Table 5.

**Table 5:** Glycemic outcomes of the included trials. TIR: time in range; TAR: time above range; HbA1c: glycated hemoglobin; AID: automated insulin delivery; AUC: area under the curve; CI: confidence interval; MRI: magnetic resonance imaging; SAP: sensor-augmented pump; pp: percentage points; mg/dL: milligrams per deciliter; mmol/mol: millimoles per mole; P: probability value. ±: plus or minus; −: minus (denoting a reduction); +: plus (denoting an increase); –: to (denoting a range); ≥: greater than or equal to; <: less than; >: greater than; %: percent.

Study	Primary endpoint	Primary endpoint result	HbA1c effect	Other key glycemic findings
Breton et al., 2020 [12]	TIR over 16 weeks	+11 pp (95% CI 7–14), P<0.001; TIR 67±10% vs 55±13%	Favored AID	Time <70 mg/dL 1.6% vs 1.8%; closed-loop mode 93%
Kariyawasam et al., 2022 [13]	Time <70 mg/dL (non-inferiority)	2.04% (95% CI 0.44–3.64) vs 7.06% (95% CI 5.46–8.66), P<0.0001	Not assessed	Non-inferiority margin of −2.5% met; superiority also shown
Ware et al., 2022 [14]	TIR over 16 weeks	+8.7 pp (95% CI 7.4–9.9), P<0.001	−0.4 pp (95% CI −0.5 to −0.3)	TAR −8.5 pp; mean glucose −12.3 mg/dL; hypoglycemia unchanged (P=0.74)
Ware et al., 2022 [15]	HbA1c at 6 months	−3.5 mmol/mol (95% CI −6.5 to −0.5), P=0.023	−0.32 pp (95% CI −0.59 to −0.04)	Closed-loop use 40% (FlorenceM) vs 93% (CamAPS FX)
von dem Berge et al., 2022 [16]	TIR	73±6% (preschool) and 69±8% (school age) on closed-loop	7.4±0.9% to 6.9±0.5%, P=0.0002	+8 and +15 pp vs SAP; >70% TIR reached by 88% and 50%
Reiss et al., 2022 [17]	Brain MRI metrics	Favored closed-loop across surface area, gray matter, and fractional anisotropy	Not significantly different	Lower full-day and night-time glucose and variability with AID
Boughton et al., 2022 [18]	C-peptide AUC at 12 months	0.35 vs 0.46 pmol/mL; difference −0.06 (95% CI −0.14 to 0.03), P=0.19	−0.4% (4 mmol/mol)	TIR 64±14% vs 54±23% (+10 pp); HbA1c <7.5% in 78% vs 56%
Messer et al., 2022 [19]	HbA1c at 13 weeks	−0.5% (95% CI −0.7 to −0.2), P<0.001	8.1±1.2% to 7.5±0.7% vs 7.8±1.1% unchanged	TIR +10 pp; baseline HbA1c ≥9.0% fell to 7.9±0.6% vs 9.8±0.8%
McVean et al., 2023 [20]	C-peptide AUC at 52 weeks	No significant between-group difference	Consistent with the TIR separation	TIR 78% vs 64% sustained to 52 weeks
Wadwa et al., 2023 [21]	TIR over 13 weeks	+12.4 pp (95% CI 9.5–15.3), P<0.001; TIR 69.3±11.1% vs 55.9±12.6%	Favored AID	Time >250 mg/dL and mean glucose improved; time <70 mg/dL unchanged

Safety and System Engagement

No trial demonstrated an increase in hypoglycemia exposure with AID, and several demonstrated a reduction. Severe hypoglycemia and diabetic ketoacidosis were uncommon throughout and were not concentrated in the intervention arms in any consistent pattern: no events of either type occurred in two trials [12,16], seven severe hypoglycemic events were distributed as four with closed-loop and three with control in a six-month trial [15], and three severe hypoglycemic events in two participants occurred with closed-loop against one with control over 24 months [18]. Diabetic ketoacidosis events, where they occurred, were consistently attributed to infusion-set failure or to non-adherent device use rather than to algorithm behavior [17,18,21]. Although diabetic ketoacidosis was uncommon in the included trials, rare overlap of diabetic ketoacidosis with the hyperosmolar hyperglycemic state at the onset of type 1 diabetes remains a distinct pediatric emergency in which hyperosmolality increases neurologic vulnerability and complicates fluid and insulin management [26]; this presentation lies outside the maintenance-therapy setting evaluated here but is relevant context for clinicians interpreting device-related ketosis. The one signal warranting attention concerned overall adverse event frequency in very young children, in whom significantly more adverse events were reported in the closed-loop arm than under standard care (60% versus 32% of participants; P=0.001), driven predominantly by infusion-set-related hyperglycemia with or without ketosis rather than by hypoglycemia [21]. Device engagement varied widely across trials, ranging from a median of 93% of time in closed-loop mode in the trial with the largest time-in-range benefit [12] to a median of 66% over 12 months in newly diagnosed adolescents [18] and to 40% with the least usable hardware configuration [15]; trials reporting higher engagement also tended to report larger glycemic effects, although this association was observed across rather than within trials and was not formally tested. Safety outcomes and engagement metrics are summarized in Table 6.

**Table 6:** Safety outcomes and system engagement across the included trials. DKA: diabetic ketoacidosis; CGM: continuous glucose monitoring; IQR: interquartile range; P: probability value. –: to (denoting a range); %: percent.

Study	Severe hypoglycemia	Diabetic ketoacidosis	System engagement	Other safety observations
Breton et al., 2020 [12]	None in either group	None in either group	Closed-loop mode median 93% (IQR 91–95)	All participants assigned to closed-loop completed the trial
Kariyawasam et al., 2022 [13]	None	None	Not reported as a headline metric	25 adverse events (18 closed-loop, 7 open loop), all treatment-related and none fatal
Ware et al., 2022 [14]	No between-group excess	None reported	High and sustained closed-loop use	Time in hypoglycemia comparable between periods (P=0.74)
Ware et al., 2022 [15]	7 events (4 closed-loop, 3 control)	2 events, both closed-loop	40% (FlorenceM) vs 93% (CamAPS FX)	155 adverse events after randomization (67 closed-loop, 88 control); 23 reportable hyperglycemia events not meeting DKA criteria
von dem Berge et al., 2022 [16]	None	None	Auto Mode 93% (preschool) and 87% (school age)	Diabetes burden and fear of hypoglycemia remained low and unchanged
Reiss et al., 2022 [17]	None reported	1 event (closed-loop, attributed to non-adherence)	Variable; some participants used the device inconsistently	No adverse effects attributable to study participation
Boughton et al., 2022 [18]	3 events in closed-loop (2 participants), 1 in control	1 event (closed-loop)	Median 66% (IQR 44–80) over 12 months	Other adverse events (34 vs 37) and serious adverse events (2 vs 4) were balanced
Messer et al., 2022 [19]	No excess with the bionic pancreas	No excess with the bionic pancreas	High device utilization throughout the 13 weeks	Adverse event profile consistent with the wider pivotal trial program
McVean et al., 2023 [20]	No safety signal precluding intensive management	No safety signal precluding intensive management	Supported by intensive contact every 1–2 weeks	Near-target glycemia was achieved without an unacceptable hypoglycemia burden
Wadwa et al., 2023 [21]	2 events (closed-loop) vs 1 (standard care)	1 event (closed-loop, infusion-set failure)	Virtual initiation successful in 55 of 68 (81%)	More adverse events in the closed-loop arm (60% vs 32% of participants; P=0.001), predominantly infusion-set-related hyperglycemia

Non-glycemic Outcomes

Three trials examined endpoints beyond glucose metrics, and their findings diverge instructively. Two independently designed trials tested the hypothesis that early, sustained near-normalization of glycemia after diagnosis would preserve residual beta-cell function, and both yielded null primary outcomes. In 97 youth aged 10.0 to 16.9 years randomized within 21 days of diagnosis, stimulated C-peptide area under the curve at 12 months did not differ between hybrid closed-loop and standard insulin therapy (geometric means 0.35 versus 0.46 pmol/mL; adjusted difference −0.06, 95% CI −0.14 to 0.03; P=0.19), despite the closed-loop group achieving 10 pp higher time in range (64±14% versus 54±23%) and 0.4% lower HbA1c [18]. In 113 youth aged 7 to 17 years with newly diagnosed type 1 diabetes, intensive management incorporating AID likewise failed to preserve C-peptide secretion at 52 weeks, although it sustained a mean time in range of 78% against 64% with standard care [20]. By contrast, a pilot randomized trial in 44 adolescents aged 14 to 17 years with early-onset disease reported that six months of hybrid closed-loop therapy produced developmental trajectories closer to those of adolescents without diabetes across cortical surface area (d = 0.74; P = 0.018), caudate volume (d = 0.75; P = 0.018), and white-matter fractional anisotropy (d = 0.68; P = 0.030), together with a gain of approximately six points in the Perceptual Reasoning Index (d = 0.82; P = 0.009) [17]. Additional outcome domains, including patient-reported measures, feasibility of remote initiation, and attainment of consensus targets, are summarized in Table 7.

**Table 7:** Synthesis of non-glycemic and implementation outcomes. AID: automated insulin delivery; HbA1c: glycated hemoglobin; TIR: time in range; IQ: intelligence quotient. <: less than; >: greater than; %: percent.

Outcome domain	Contributing studies	Direction	Principal message
Residual beta-cell function	Boughton et al., 2022 [18]; McVean et al., 2023 [20]	Neutral	Neither 12 months of closed-loop therapy nor 52 weeks of AID-supported intensive management preserved stimulated C-peptide; AID should not be offered for disease modification
Neurodevelopment and cognition	Reiss et al., 2022 [17]	Favorable	More neurotypical brain development and a gain of about six IQ points, from a small proof-of-concept pilot requiring replication
Patient- and family-reported outcomes	von dem Berge et al., 2022 [16]; Boughton et al., 2022 [18]	Neutral to favorable	Diabetes burden, hypoglycemia fear, and quality of life did not deteriorate, but trials were underpowered and used heterogeneous instruments
Remote initiation	Wadwa et al., 2023 [21]	Favorable	81% of closed-loop initiations were completed virtually without loss of efficacy, supporting decentralized onboarding
Benefit at high baseline HbA1c	Ware et al., 2022 [15]; Messer et al., 2022 [19]	Favorable	Absolute gains were largest in those furthest from target, provided device engagement was high
Attainment of consensus targets	von dem Berge et al., 2022 [16]; Boughton et al., 2022 [18]; McVean et al., 2023 [20]	Favorable	Most preschoolers exceeded 70% TIR, and 78% versus 56% of newly diagnosed youth reached HbA1c <7.5% at 12 months

Risk of Bias

Seven of the ten trials were judged to be at low risk of bias overall, and the remaining three raised some concerns; no trial was rated at high risk of bias in any domain. Concerns regarding the randomization process (domain 1) applied to two trials: a very small crossover trial in which allocation concealment was incompletely described [13], and a pilot trial that used simple unstratified randomization with only six to 13 participants recruited per site [17]. Concerns regarding deviations from intended interventions (domain 2) applied to the same two trials and additionally to the 24-month trial in newly diagnosed youth [18], in which the combination of open-label allocation and a lengthy follow-up period created scope for co-intervention and for crossover to pump therapy within the control arm. Missing outcome data (domain 3) raised concerns only in that trial, reflecting incomplete availability of mixed-meal tolerance test data at the 12-month primary endpoint, with differential availability between arms. Measurement of the outcome (domain 4) was judged at low risk throughout, because the principal endpoints were derived from continuous glucose monitoring, centrally analyzed glycated hemoglobin, or blinded imaging and cognitive assessment, none of which is susceptible to appreciable observer bias. Selection of the reported result (domain 5) raised concerns only in the pilot neurodevelopmental trial [17], in which numerous imaging endpoints were examined without a prespecified analytical hierarchy.

**Figure 4:**
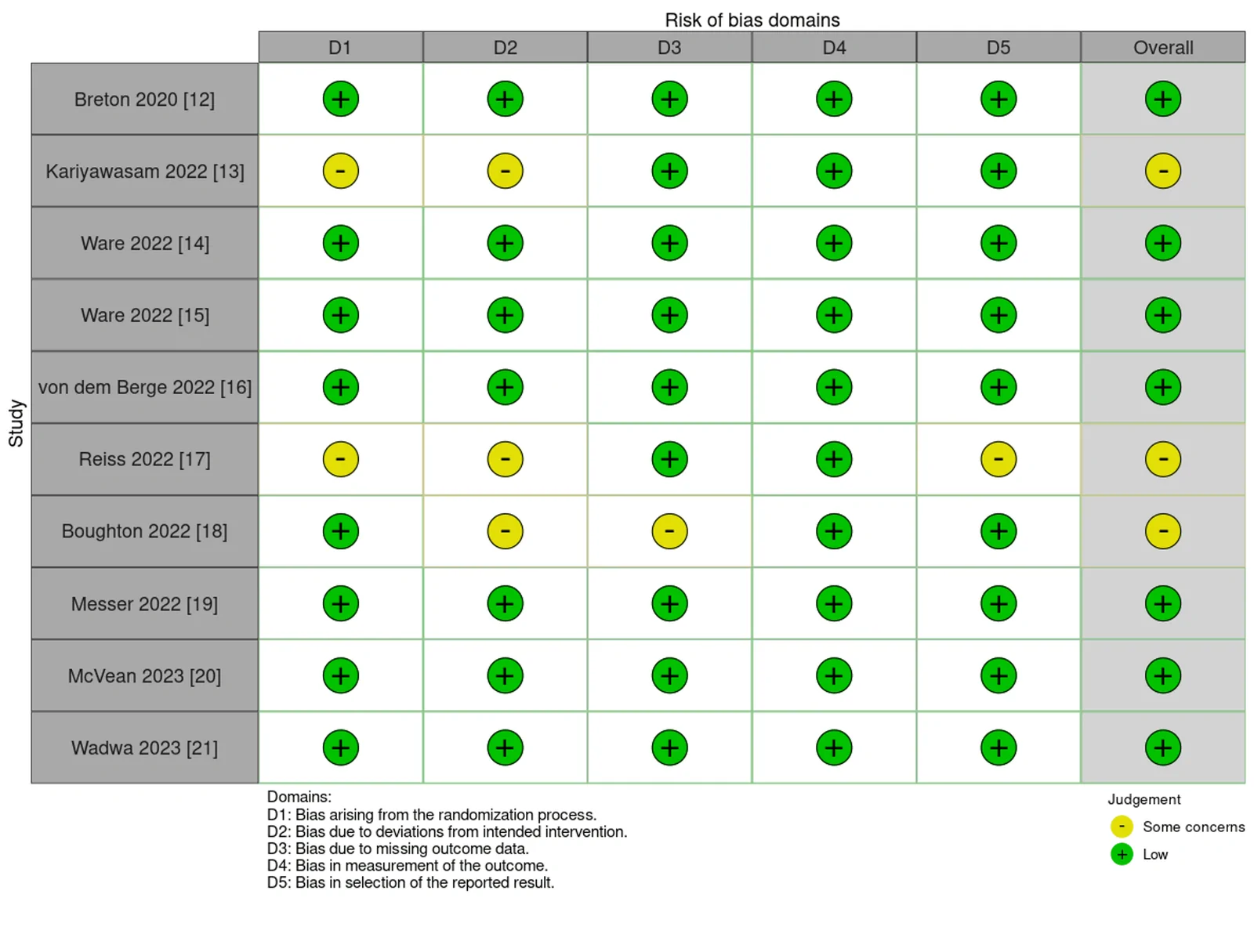
Risk-of-bias assessment of the included trials using the Cochrane RoB 2 tool. Green plus signs denote a low risk of bias, and yellow minus signs denote some concerns; no domain in any trial was judged to be at high risk of bias. Each trial was assessed against its own primary endpoint, as specified in Table 5. Domains: D1: bias arising from the randomization process; D2: bias due to deviations from intended interventions; D3: bias due to missing outcome data; D4: bias in measurement of the outcome; D5: bias in selection of the reported result. RoB 2: revised Cochrane risk-of-bias tool for randomized trials. +: low risk of bias; −: some concerns.

It is worth noting that open-label allocation is unavoidable when a visible pump-and-algorithm system is compared with injections or with a non-automated pump, and it was therefore not treated as sufficient in itself to downgrade a trial. Every included trial analyzed participants according to their randomized assignment, which preserves an unbiased estimate of the effect of assignment to the intervention. Domain-level and overall judgments are displayed in Figure 4.

Discussion

Principal Findings

This systematic review of ten randomized controlled trials enrolling 888 children and adolescents indicates that automated insulin delivery improves glycemic control across the entire pediatric age spectrum, from infancy through late adolescence, without an accompanying increase in hypoglycemia. Where time in range was the primary endpoint, between-group differences clustered between 8.7 and 12.4 pp, corresponding to roughly two to three additional hours per day within the target range [12,14,21]. Where HbA1c was the primary endpoint, reductions of 0.32% to 0.5% favored AID [15,19]. The consistency of direction across five different control algorithms, four comparator regimens, and intervention durations spanning six weeks to two years is the single most persuasive feature of this evidence base, because it suggests that the benefit derives from the principle of algorithmic insulin modulation rather than from any particular commercial implementation.

Three findings deserve emphasis because they are not adequately captured by a summary effect estimate. The first concerns age. It was widely anticipated that very young children would benefit least from AID, given their small insulin doses, extreme day-to-day variability in requirements, unpredictable eating, and near-total dependence on caregivers. The available trial evidence suggested the opposite: the largest time-in-range difference in this review was observed in children aged 2 to under 6 years [21], and a crossover trial spanning both preschool and school-aged strata found that preschoolers achieved the consensus target of spending more than 70% of time in range considerably more often than their older counterparts [16]. A plausible explanation is that the sources of variability that make this age group difficult to manage manually are precisely those that an algorithm operating every five minutes is well suited to accommodate, whereas older children introduce behavioral variability-missed boluses, deliberate under-treatment, competing adolescent priorities-that no basal-modulating algorithm can fully compensate for. These findings challenge the assumption that very young children derive less benefit from AID, and argue against age-based restrictions on access. It should be emphasized that this comparison is between trials rather than within them; no included trial randomized across age strata, and the observation is therefore hypothesis-generating.

The second finding concerns engagement, which emerged as the factor most consistently associated with the size of the observed effect. The trial reporting the smallest HbA1c difference was also the trial in which one hardware configuration achieved a median of only 40% closed-loop use because of failing phone enclosures, against 93% for the alternative configuration within the same trial [15]. Conversely, the trials reporting the largest glycemic separations were those in which the system was engaged for more than 90% of the time [12,16]. The intermediate level of engagement recorded over 12 months in newly diagnosed adolescents [18] sits between these poles and may help explain why the glycemic separation in that trial, while real, was smaller than in shorter studies. Because no formal interaction test or meta-regression was performed, engagement should be regarded as a candidate rather than an established effect modifier. The practical implication nonetheless follows: the efficacy demonstrated in these trials is contingent rather than automatic, and hardware reliability, alarm burden, form factor, and structured onboarding are not peripheral usability concerns but plausibly direct determinants of clinical outcome.

Comparison With Previous Literature

These observations align with the pooled pediatric evidence, in which a meta-analysis of 25 outpatient trials in children and adolescents reported an 11.38 pp increase in time in range (95% CI 9.01-13.76) equivalent to 164 minutes per day, with the effect consistent whether AID was used over three or six months [27]. A more recent meta-analysis restricted to youth aged 6 to 18 years and including 11 trials likewise found clinically meaningful improvements across multiple glucose metrics, most pronounced overnight and without an increase in adverse events, while noting that the effect on quality of life remains unclear [28]. The concordance between our narrative estimates and these pooled analyses is reassuring, and readers seeking a single summary figure are directed to them.

Our findings are also consistent with, and extend, evidence from trials in adolescents and young adults that fell outside our age criterion and are tabulated in Table 3. The FLAIR trial, comparing two hybrid closed-loop systems in 113 participants aged 14 to 29 years, demonstrated that algorithm generation itself matters, with the advanced system reducing hyperglycemia without increasing hypoglycemia relative to its predecessor [22]. A six-month Australian trial in 135 children and adolescents reported a mean adjusted time-in-range difference of 6.7 pp with hybrid closed-loop compared with conventional therapy, alongside improved diabetes-specific quality of life [23]; the smaller glycemic effect in that trial, which enrolled participants aged 12 to 25 years, is consistent with our observation that effect size diminishes as adolescent behavioral variability increases. A subsequent trial by the same group, identified in our updated search and the most recent randomized evidence in this population, randomized youth aged 12 to 25 years with HbA1c above 8.5% to advanced hybrid closed-loop therapy or treatment as usual for six months and reported a mean adjusted between-group HbA1c difference of −0.77%, without accompanying improvement in psychosocial outcomes [24]. That trial is important for two reasons: it confirms in a randomized design that the largest absolute glycemic gains occur in those furthest from target, mirroring the gradient we observed [15,19]; and it cautions that glycemic improvement does not automatically translate into reduced diabetes distress, reinforcing the need for psychological support alongside device provision. Finally, a multicenter trial in 122 prepubertal children compared 24-hour closed-loop activation with evening-and-night-only activation and found the round-the-clock configuration superior, establishing that the daytime period contributes materially to the overall benefit and that partial automation is not an adequate substitute [25]. Collectively, these adjacent trials reinforce the core conclusions of this review while indicating that the transition through adolescence is the point at which technological benefit is most readily eroded.

What Automated Insulin Delivery Does Not Achieve

The third principal finding concerns the limits of the technology. Two well-designed trials tested whether early, sustained near-normalization of glucose after diagnosis preserves residual beta-cell function, and both yielded null primary outcomes despite achieving substantial glycemic separation [18,20]. The 12-month C-peptide result in the CLOuD (Closed Loop from Onset in Type 1 Diabetes) trial was unchanged in a per-protocol analysis restricted to participants with at least 60% closed-loop use, and the CLVer (Hybrid Closed Loop Therapy and Verapamil for Beta Cell Preservation in New Onset Type 1 Diabetes) trial sustained a mean time in range of 78% for a full year without detectable benefit to C-peptide [18,20]. Extended follow-up to 48 months in the CLOuD cohort confirmed that the decline in C-peptide was not slowed, while also demonstrating that glycemic control in the closed-loop group was maintained over four years [29]. These trials establish that intensified glycemic control delivered by AID does not measurably slow the loss of stimulated C-peptide over one to four years after diagnosis. They do not directly test the mechanisms underlying beta-cell loss, and we therefore draw only the inference the data support: whatever the contribution of glucotoxicity to beta-cell decline, it is not sufficiently modifiable by glycemic control at the level achieved in these trials to alter the observed trajectory. The clinical implication is nonetheless clear. AID should be prescribed for glycemic and quality-of-life benefit rather than in the expectation of disease modification, and strategies aimed at preserving beta-cell function will need to be evaluated on their own terms rather than as a secondary hope attached to glucose management.

Set against this, the neurodevelopmental signal reported in adolescents is the most provocative result in the review [17]. That six months of closed-loop use was associated with brain structural and functional trajectories closer to neurotypical development, and with a gain of roughly six points in a standardized index of nonverbal reasoning, provides the first randomized evidence that the cognitive consequences of chronic hyperglycemia in childhood might be modifiable rather than fixed. The finding is biologically coherent with observational work demonstrating divergent gray and white matter trajectories in children with type 1 diabetes that correlate with cumulative hyperglycemia [3]. It must nonetheless be interpreted with caution: the trial was explicitly a proof-of-concept pilot with 21 participants per arm, examined numerous imaging endpoints, and detected no between-group difference in HbA1c, so the mechanistic pathway from device to brain remains inferential. Replication in an adequately powered trial with longer follow-up is a clear priority, and would be of considerably greater consequence for families than a further increment in time in range.

Knowledge Gaps and Implications for Practice

Several knowledge gaps follow from this synthesis. First, all included trials were conducted in high-income settings in North America [12,15,17,19-21], western Europe [13,14,16], and the United Kingdom [14,15,18], and their participants were predominantly of white ethnicity with high pre-existing rates of pump and sensor use. Given that a fifth of the global type 1 diabetes population lives in low-income and lower-middle-income countries [1], the external validity of this evidence base is narrow, and no randomized evidence addresses AID deployment in resource-constrained health systems. Second, follow-up remains short relative to the duration of childhood: only two trials extended beyond six months, and none reported microvascular, growth, or bone outcomes. Third, patient-reported outcomes were assessed inconsistently and with heterogeneous instruments, leaving the effect of AID on family burden, sleep, and diabetes distress poorly quantified despite these being the outcomes families most often cite; the recent finding that a substantial HbA1c improvement occurred without measurable psychosocial gain [24] makes this gap more pressing rather than less. Fourth, no trial systematically evaluated discontinuation, even though real-world attrition from closed-loop therapy is well documented, and the trial populations were by construction enriched for participants willing to use technology. Fifth, the feasibility of remote initiation demonstrated in one trial [21] opens a promising avenue for widening access that has not yet been formally tested as a randomized implementation strategy.

A final consideration concerns how these trial results should be translated into service delivery. Every included trial embedded its intervention within a research infrastructure that provided structured education, frequent contact, and rapid technical support, and in one trial the intervention arm received study-team contact every one to two weeks [20]. Such intensity is not reproducible in most routine pediatric diabetes services, which raises the possibility that effectiveness in ordinary care will fall short of the efficacy reported here unless commensurate support is funded alongside the devices themselves. The corollary is that the observed dependence of benefit on device engagement should be read not as a statement about patient adherence but as a statement about system design: engagement is produced by reliable hardware, tolerable alarm burden, and accessible clinical support, all of which are modifiable at the level of health policy. Demonstrating that four in five closed-loop initiations can be completed remotely without loss of glycemic benefit [21] suggests that at least part of this support can be delivered at a distance, which is directly relevant to the geographical and workforce constraints that currently limit pediatric access.

Limitations

This review has strengths and limitations. Its strengths include a prespecified protocol, duplicate screening, extraction and risk-of-bias assessment, a search spanning five sources with citation tracking, and a deliberately restrictive population criterion that excluded mixed adult-pediatric cohorts and thereby avoided the dilution that affects several previous syntheses. Restricting comparators to non-automated therapy also ensured that every included estimate addresses the clinically relevant question of whether to initiate AID, rather than which AID system to select. Several limitations should be weighed against these strengths. The protocol was not prospectively registered, which limits external verification that the eligibility criteria, including the conditional recency restriction, were specified in advance. The principal analytical limitation is the absence of meta-analysis; as set out in the Methods, the included trials did not share a common estimand, and a pooled estimate would have obscured the age and engagement gradients that constitute the most clinically actionable observations, but this decision necessarily forgoes a single summary effect and precludes formal assessment of publication bias or of subgroup interaction. Related to this, the differences observed across age strata and levels of device engagement are between-study comparisons that are vulnerable to confounding by trial-level factors such as comparator choice, duration, and calendar year, and should be treated as hypothesis-generating. Further limitations include restriction to English-language publications, the exclusion of trials enrolling mixed adolescent-young adult cohorts that carry information about the upper end of the pediatric age range, the modest size of several included trials, and the fact that three trials raised some concerns for bias, chiefly relating to the randomization process, co-intervention during long open-label follow-up, and incomplete outcome data. Industry provision of devices and consumables was common, although funding in most trials came from public or charitable sources.

## Conclusions

Randomized evidence generated exclusively in children and adolescents indicates that automated insulin delivery systems consistently improve glycemic control compared with non-automated insulin therapy, delivering between-group increases in time in range of approximately 9 to 12 percentage points and HbA1c reductions of approximately 0.3% to 0.5%, without increasing hypoglycemia. Benefit extends across the pediatric age spectrum and, contrary to prior expectation, was largest in the youngest children, in whom manual management is most difficult. The magnitude of benefit was most consistently associated with sustained device engagement, which makes hardware reliability, usability, and structured onboarding clinical rather than merely technical considerations, although this association was observed across trials and requires formal testing. Automated insulin delivery does not preserve residual beta-cell function and should not be offered on that basis, whereas preliminary randomized evidence that it may support more typical brain development in adolescents merits replication. These findings support automated insulin delivery as the default insulin delivery modality for children and adolescents with type 1 diabetes wherever it can be provided and sustained, and they identify equitable access, long-term outcomes, and rigorous measurement of family-reported burden as the priorities for the next generation of trials.

## References

[1] Gregory GA, Robinson TIG, Linklater SE (2022). Global incidence, prevalence, and mortality of type 1 diabetes in 2021 with projection to 2040: a modelling study. Lancet Diabetes Endocrinol.

[2] Nathan DM, Genuth S, Lachin J (1993). The effect of intensive treatment of diabetes on the development and progression of long-term complications in insulin-dependent diabetes mellitus. N Engl J Med.

[3] Mauras N, Buckingham B, White NH (2021). Impact of type 1 diabetes in the developing brain in children: a longitudinal study. Diabetes Care.

[4] Battelino T, Danne T, Bergenstal RM (2019). Clinical targets for continuous glucose monitoring data interpretation: recommendations from the international consensus on time in range. Diabetes Care.

[5] Biester T, Berget C, Boughton C (2024). International Society for Pediatric and Adolescent Diabetes clinical practice consensus guidelines 2024: diabetes technologies - insulin delivery. Horm Res Paediatr.

[6] Adolfsson P, Hanas R, Zaharieva DP, Dovc K, Jendle J (2024). Automated insulin delivery systems in pediatric type 1 diabetes: a narrative review. J Diabetes Sci Technol.

[7] Phillip M, Nimri R, Bergenstal RM (2023). Consensus recommendations for the use of automated insulin delivery technologies in clinical practice. Endocr Rev.

[8] Page MJ, McKenzie JE, Bossuyt PM (2021). The PRISMA 2020 statement: an updated guideline for reporting systematic reviews. BMJ.

[9] Sterne JA, Savović J, Page MJ (2019). RoB 2: a revised tool for assessing risk of bias in randomised trials. BMJ.

[10] McGuinness LA, Higgins JP (2021). Risk-of-bias VISualization (robvis): An R package and Shiny web app for visualizing risk-of-bias assessments. Res Synth Methods.

[11] Campbell M, McKenzie JE, Sowden A (2020). Synthesis without meta-analysis (SWiM) in systematic reviews: reporting guideline. BMJ.

[12] Breton MD, Kanapka LG, Beck RW (2020). A randomized trial of closed-loop control in children with type 1 diabetes. N Engl J Med.

[13] Kariyawasam D, Morin C, Casteels K (2022). Hybrid closed-loop insulin delivery versus sensor-augmented pump therapy in children aged 6-12 years: a randomised, controlled, cross-over, non-inferiority trial. Lancet Digit Health.

[14] Ware J, Allen JM, Boughton CK (2022). Randomized trial of closed-loop control in very young children with type 1 diabetes. N Engl J Med.

[15] Ware J, Boughton CK, Allen JM (2022). Cambridge hybrid closed-loop algorithm in children and adolescents with type 1 diabetes: a multicentre 6-month randomised controlled trial. Lancet Digit Health.

[16] von dem Berge T, Remus K, Biester S (2022). In-home use of a hybrid closed loop achieves time-in-range targets in preschoolers and school children: results from a randomized, controlled, crossover trial. Diabetes Obes Metab.

[17] Reiss AL, Jo B, Arbelaez AM (2022). A pilot randomized trial to examine effects of a hybrid closed-loop insulin delivery system on neurodevelopmental and cognitive outcomes in adolescents with type 1 diabetes. Nat Commun.

[18] Boughton CK, Allen JM, Ware J (2022). Closed-loop therapy and preservation of C-peptide secretion in type 1 diabetes. N Engl J Med.

[19] Messer LH, Buckingham BA, Cogen F (2022). Positive impact of the bionic pancreas on diabetes control in youth 6-17 years old with type 1 diabetes: a multicenter randomized trial. Diabetes Technol Ther.

[20] McVean J, Forlenza GP, Beck RW (2023). Effect of tight glycemic control on pancreatic beta cell function in newly diagnosed pediatric type 1 diabetes: a randomized clinical trial. JAMA.

[21] Wadwa RP, Reed ZW, Buckingham BA (2023). Trial of hybrid closed-loop control in young children with type 1 diabetes. N Engl J Med.

[22] Bergenstal RM, Nimri R, Beck RW (2021). A comparison of two hybrid closed-loop systems in adolescents and young adults with type 1 diabetes (FLAIR): a multicentre, randomised, crossover trial. Lancet.

[23] Abraham MB, de Bock M, Smith GJ (2021). Effect of a hybrid closed-loop system on glycemic and psychosocial outcomes in children and adolescents with type 1 diabetes: a randomized clinical trial. JAMA Pediatr.

[24] Abraham MB, Smith GJ, Dart J (2025). Glycemic and psychosocial outcomes of advanced hybrid closed-loop therapy in youth with high HbA1c: a randomized clinical trial. Diabetes Care.

[25] Renard E, Tubiana-Rufi N, Bonnemaison E (2022). Outcomes of hybrid closed-loop insulin delivery activated 24/7 versus evening and night in free-living prepubertal children with type 1 diabetes: a multicentre, randomized clinical trial. Diabetes Obes Metab.

[26] Filippatos F, Themelis G, Dolianiti M, Kanaka-Gantenbein C, Kakleas K (2026). Case report of overlap of diabetic ketoacidosis and hyperosmolar hyperglycemic state in a 5-year-old with new-onset type 1 diabetes mellitus: diagnostic and management considerations. Reports (MDPI).

[27] Zeng B, Gao L, Yang Q, Jia H, Sun F (2023). Automated insulin delivery systems in children and adolescents with type 1 diabetes: a systematic review and meta-analysis of outpatient randomized controlled trials. Diabetes Care.

[28] de Visser HS, Waraich S, Chhabra M (2025). Automated insulin delivery systems and glucose management in children and adolescents with type 1 diabetes: a systematic review and meta-analysis. JAMA Pediatr.

[29] Ware J, Boughton CK, Allen JM (2024). Effect of 48 months of closed-loop insulin delivery on residual C-peptide secretion and glycemic control in newly diagnosed youth with type 1 diabetes: a randomized trial. Diabetes Care.

